# Effectiveness of Individualized Training Programs Based on the Optimal Force-Velocity Relationship to Develop Athletes’ Jump Performance: A Systematic Review with Meta-Analysis

**DOI:** 10.5114/jhk/201963

**Published:** 2025-09-23

**Authors:** Zhaoqian Li, Peng Zhi, Xing Zhang, Junbei Bai, Amador García-Ramos, Danica Janicijevic

**Affiliations:** 1 Department of Physical Education and Sport, Faculty of Sport Sciences, University of Granada, Granada, Spain.; 2 Department of Physiotherapy, University of Melbourne, Melbourne, Australia.; 3 Competitive Sports, Beijing Research Institute of Sports Science, Beijing, China.; 4 Department of Sports Sciences and Physical Conditioning, Faculty of Education, Catholic University of the Most Holy Conception, Concepción, Chile.; 5 Department of Radiology, Ningbo No. 2 Hospital, Ningbo, China.; 6 Faculty of Sports Science, Ningbo University, Ningbo, China.

**Keywords:** athletes, resistance training, imbalance, countermovement jump, team sports

## Abstract

This systematic review with meta-analysis evaluated whether individualized training programs tailored to an athlete’s F-V profile were more effective than non-individualized programs (i.e., without considering the athlete´s F-V profile) in decreasing F-V imbalances and enhancing jump height and maximal power (P_max_). A literature search was conducted in PubMed, Web of Science, EBSCO and Cochrane Library databases from inception until April 19^th^ 2024. Pooled meta-analysis and subgroup meta-analysis were performed using the random-effects and fixed-effects models. The individualized training program was more effective at reducing the F-V imbalance compared to the non-individualized training program (SMD = 0.59 [95%, p < 0.001), but no significant differences were reached for jump height (SMD = 0.50, p = 0.059) and P_max_ (SMD = 0.10, p = 0.543). Regarding subgroup analyses, differences were found only between the velocity-deficit subgroup and the non-individualized group with the former showing greater reductions in the F-V imbalance (SMD = 1.28, p < 0.001) and greater improvements in jump height (SMD = 0.77, p = 0.010), but no significant differences were noted for P_max_ (SMD = 0.40, p = 0.165). No significant differences in the F-V imbalance, jump height or P_max_ were obtained between force-deficit and well-balanced subgroups compared to the non-individualized group. Individualized training programs are more effective at reducing F-V imbalances than non-individualized programs because they target specific segments of the F-V profile. However, pooled evidence suggests that individualized training is only more effective at enhancing jump height for athletes experiencing a velocity-deficit at the start of the training program.

## Introduction

Vertical jumping refers to a type of physical activity where individuals propel themselves off the ground in an upward direction using the lower limb muscles (Samozino et al., 2014a). The two most common types of vertical jumps include the squat jump (SJ) which requires holding in a squat position briefly before jumping, and the countermovement jump (CMJ), starting upright and quickly transitioning to a semi-squat before jumping (Bobbert et al., 1996; Van Hooren and Zolotarjova, 2017). The CMJ is frequently favored for assessing athletic performance as it is regarded as a more specific and natural movement (Kozinc et al., 2022), while the SJ is the preferable choice when assessing the rate of force development (Harris et al., 2008; Köklü et al., 2015). Jump height significantly impacts sport-specific performance, such as the success rate of jumping shots in basketball and the efficacy of spike and block manoeuvres in volleyball (Nishiumi and Hirose, 2024; Okazaki et al., 2015; Riemann et al., 2024; Ziv and Lidor, 2010). Therefore, enhancing jump performance is important for improving performance in specific sports and continues to be a valuable area of focus in athletic training (Cronin and Sleivert, 2005; Jiménez-Reyes et al., 2016).

Many different training programs have been implemented to maximize athletes’ jumping performance (García-Ramos et al., 2018; Sáez de Villarreal et al., 2013). One of the most recent proposals relies on the information obtained from vertical jump specific force-velocity (F-V) profiling, a novel methodology used for identifying force and velocity deficits during the vertical jumping performance of athletes and individualizing training programs accordingly. Specifically, Samozino et al. (2012) pointed out that for particular maximal power output (P_max_), there was an optimal slope of the F-V profile (i.e., the ratio between the maximal theorical force [*F_0_*] and maximal theoretical velocity [*v_0_*], derived as the intercept from the unloaded and loaded vertical jump test) that allowed reaching maximal jump height. For example, if two athletes exhibit comparable P_max_, the athlete whose F-V profile aligns more closely with the optimal slope is likely to achieve greater jump height compared to the athlete whose F-V profile deviates from the optimal slope (Morin and Samozino, 2016). Meanwhile, the theory demonstrates that a force deficit or a velocity deficit may limit an athlete's vertical jump height by up to 20% (Samozino et al., 2014a). Therefore, athletes with smaller deficits tend to achieve greater jump height generally. Another advantage of using F-V profiles to enhance jump height is the simplicity of the assessment protocols. Determining jump height and F-V variables necessitate performing only two jumps (one unloaded and one with a submaximal load), and this can be efficiently achieved using smartphone applications such as My Jump (Balsalobre-Fernández et al., 2015; García-Ramos et al., 2021; Janicijevic et al., 2020; Yingling et al., 2018).

It is not surprising that using F-V profiling to enhance jump height has piqued the curiosity of sports scientists due to its simplicity and potential informational value. Many studies have begun investigating whether the training programs tailored based on individual F-V profiles (i.e., individualized training programs) could enhance jump height more effectively than training programs that do not consider individual F-V profiles (i.e., non-individualized training programs) (Barrera-Domínguez et al., 2023; Jiménez-Reyes et al., 2017; Lindberg et al., 2021; Simpson et al., 2021; Zabaloy et al., 2020). The uniqueness of training programs based on the individual F-V profiles lies in applying different loading conditions for athletes with a velocity deficit (i.e., the actual F-V slope higher than the optimal F-V slope) compared to those with a force deficit (i.e., the actual F-V slope lower than the optimal F-V slope). For instance, light (e.g., < 30% of 1RM) and negative loads are implemented when athletes experience a velocity deficit, while heavy loads (e.g., > 80% of 1RM) are used by athletes with a force deficit (Alcazar et al., 2021; Escobar Álvarez et al., 2020; Jiménez-Reyes et al., 2017). Some longitudinal studies supported the effectiveness of training programs based on individual F-V profiles to mitigate F-V imbalances and improve jump height (Barrera-Domínguez et al., 2023; Jiménez-Reyes et al., 2017; Simpson et al., 2021). However, other studies questioned the superiority of individualized training over non-individualized training programs (Lindberg et al., 2021; Zabaloy et al., 2020). The relative novelty of individualized training programs based on the F-V profile, combined with the lack of a comprehensive review comparing their effectiveness to non-individualized programs, makes it challenging to determine which approach is superior.

It is also important to emphasize that most studies primarily explored the differences between individualized and non-individualized training programs, without thoroughly examining the subtle distinctions within the individualized program that aimed to reduce force versus velocity deficits (Barrera-Domínguez et al., 2023; Jiménez-Reyes et al., 2019; Lindberg et al., 2021). It should be noted that the effectiveness of individualized training programs might also depend on the magnitude of the force and velocity deficits. For instance, Jimenez-Reyes et al. (2017) reported similar effect sizes (1.00 vs. 0.93) for the increase in jump height in both force-deficit and velocity-deficit subgroups following an individualized training program. However, two years later the same authors suggested that the force-deficit subgroup benefited more in decreasing the F-V imbalance and enhancing jump height than the velocity-deficit subgroup (Jiménez-Reyes et al., 2019). A possible reason for this discrepancy might be that the study conducted before (Jiménez-Reyes et al., 2017) had fixed intervention duration, whereas the later study (Jiménez-Reyes et al., 2019) did not have a set timeframe, allowing training to conclude once the F-V imbalance was corrected. Hence, besides comparing purely individualized vs. non-individualized training groups, it would be important to compare the effectiveness of the different individualized training subgroups (i.e., force-deficit and velocity-deficit) against the non-individualized group in decreasing F-V imbalances and jump performance enhancement.

Therefore, the primary aim of this systematic review and meta-analysis was to compare the effectiveness of individualized and non-individualized training programs in decreasing F-V imbalances and enhancing jump height and P_max_. Our secondary aim was to elucidate which subgroup could benefit more from individualized training programs, i.e., the force-deficit, the velocity-deficit or the well-balanced training group. We hypothesized that individualized training programs would be more effective in mitigating F-V imbalances, and consequently in improving jump height and P_max_. We could not establish a hypothesis regarding the superiority of any specific subgroup in decreasing F-V imbalances and improving jumping performance due to discrepancies found in the literature.

## Methods

### Study Selection

The PICO strategy was used for selecting the articles (participants [healthy individuals], intervention [individualized training programs based on the F-V profile], comparison [individualized vs. non-individualized training programs], and outcome [F-V imbalance, jump height, and P_max_]). For the systematic review, only studies that (I) included individualized training programs and non-individualized training programs, (II) underwent a peer review process, and (III) were written in English, were considered. Individualized training programs involved categorizing subjects into force-deficit, velocity- deficit and well-balanced training subgroups based on their F-V imbalances, which formed the basis for the subgroup analyses.

Study eligibility was assessed independently by two authors (Z.L. and P.Z.). All records were imported into EndNote X8 (Clarivate Analytics, Philadelphia, PA, USA), and duplicates were removed based on the author(s), the title, and the publication year. Titles and abstracts were then screened to determine initial eligibility. Subsequently, full texts of the remaining records were retrieved and assessed for eligibility. Any inconsistencies encountered during the study selection process were addressed through discussion between the two authors. If necessary, a third author provided judgment to resolve any discrepancies (X.Z.). The current systematic review and meta-analysis adhered to the guidelines outlined in the Cochrane Handbook for Systematic Reviews of Interventions, version 5.1.0, and followed the Preferred Reporting Items for Systematic Reviews and Meta-Analysis (PRISMA) 2020 checklist (Page et al., 2021).

### Information Sources and Search Strategy

A systematic literature search was performed using the following electronic databases: PubMed, Web of Science, EBSCO, and Cochrane Library. The search period extended from the inception of each database until April 19^th^ 2024. The following syntax was adapted for each database and applied to the title, abstract, and keyword search fields: ([individualized OR optimized] AND [training] AND [force-velocity]). During the subsequent phase of the search, the reference lists of review studies identified in the initial search were screened. Studies that met the inclusion criteria were further investigated by searching for “similar studies” through Google Scholar. Conference abstracts and proceedings were not considered.

### Data Collection and Analysis

From the studies that met the inclusion criteria, the following data were extracted (1) study identification information, (2) study design, (3) sample size, (4) participants’ age and sex, (5) exercise used to establish the F-V profile (SJ or CMJ), (6) training program information, and (7) means and standard deviations for pre and post F-V variables (P_max_ and F-V imbalance) and jump height. When authors presented results exclusively in figures, GetData Graph Digitizer 2.26 software (GetData Software Pty Ltd, Kogarah, NSW, Australia) was utilized to extract the data. When original studies did not provide sufficient data, authors were contacted via e-mail. The data extraction process was independently conducted by two authors (Z.L. and P.Z.). Any discrepancies during data collection were resolved through discussion between the two authors or, if necessary, by the judgment of a third author (X.Z.).

### Quality of the Study and the Risk of Bias

The quality of each study was independently assessed by two authors (Z.L. and P.Z.), and discrepancies were resolved together through discussion or by help of a third author (X.Z.). The quality and risk of bias were evaluated using the Cochrane Risk of Bias tool (Higgins et al., 2019), where five domains of bias are assessed (i.e., randomization process, deviations from intended interventions, missing outcome data, measurement of the outcome, and selection of the reported result). A value of high, low risk, and some concerns were provided for each domain.

### Statistical Analysis

Due to the inconsistency in the data calculation methods and the presence of subgroup data provided by some experiments meeting the screening criteria, necessary pretreatment needed to be finished a priori. For studies providing only subgroup data, mathematical computations were utilized to merge these subgroups into a combined one as an individualized training program or a non-individualized training program for further analyses. Additionally, some studies used the percentages of the F-V imbalance (%FV_imb_) from the current profile to the optimal profile, with 0% indicating a perfectly balanced F-V profile, while other studies used absolute difference between the current profile to optimal (%FV_opt_) where 100% implied a perfectly balanced F-V profile. The conversion from differences between pre- and post-intervention %FV_imb_ to %FV_opt_ was calculated using the following equation (1). In our case, only %FV_opt_ was used as a F-V imbalance for further analyses.


$$\Delta \%FV_{opt}=\%FVimb_{pre}-\%FVimb_{post}\;\left(1\right)$$

Dependent variables were obtained in the form of mean values and standard deviations (SDs). Training effects of F-V variables (F-V imbalance, P_max_) and jump height were determined by subtracting the pre-intervention mean value from the post-intervention mean value for both the individualized and non-individualized training groups. The SD of the training effect was calculated using the pre-intervention and post-intervention SDs according to formula (2) and assumed a conservative correlation coefficient of 0.5 (Zhang et al., 2023).


$$SD_{change}=\sqrt{SD_{pre}^{2}}+SD_{post}^{2}-2\times 0.5\times SD_{pre}SD_{Post}$$

Fixed effect models using the inverse-variance method were performed for the dependent variables with heterogeneity below 50% and random effect models using the inverse-variance method for the dependent variables with heterogeneity higher than 50%. As no subgroup data were reported in some studies (Barrera-Domínguez et al., 2023; Simpson et al., 2021), additional subgroup fixed-effects or random models were conducted in the remaining studies to compare different subgroups (force-deficit training, velocity-deficit training, well-balanced training) with the non-individualized training program. Pooled estimates of the effect size obtained through either comprehensive or subgroup meta-analyses were presented as standardized mean differences (SMD) with 95% confidence intervals (95% CI). Statistical significance was considered at *p* ≤ 0.05. The interpretation scale for effect size magnitude used in training research was as follows: negligible (<0.2), small (0.2–0.5), moderate (0.5–0.8), and large (≥0.8) (García-Ramos et al., 2018). The *I*^2^ statistic represented the percentage of total variation in estimated effects across studies due to heterogeneity rather than chance, and *I*^2^ ≥ 50% was regarded as high heterogeneity (Zhao et al., 2016). Data were analysed using Stata 17 software (StataCorp LLC, Texas, US).

## Results

### Search Results

**Figure 1:**
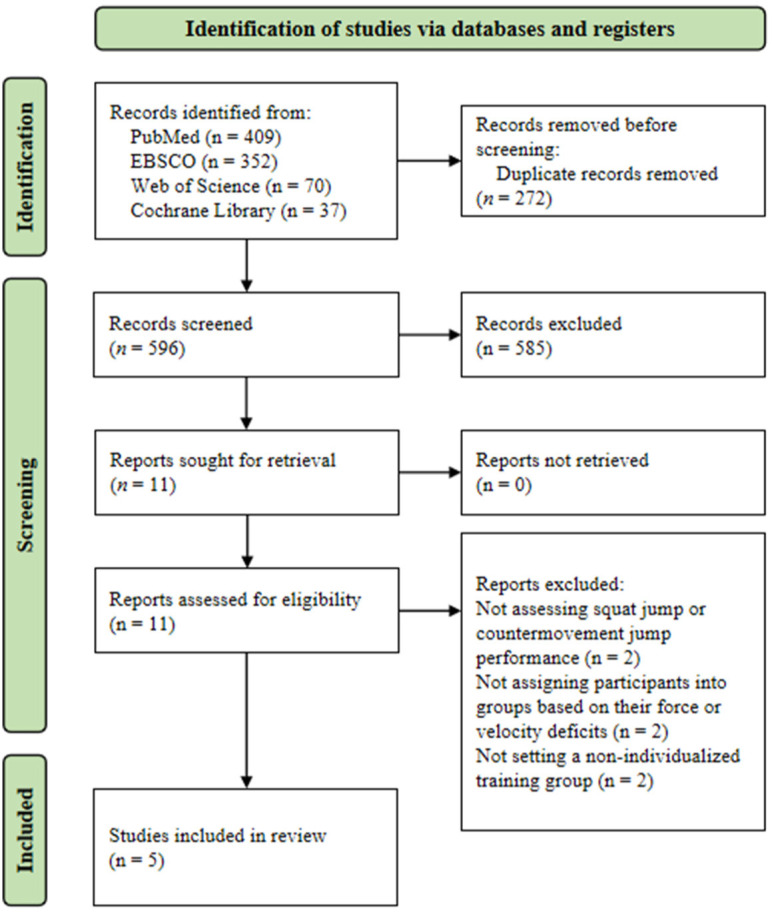
Flowchart of the study selection for interventions comparing changes in force-velocity (F-V) imbalance, jump height, and maximal power between individualized training programs (based on the F-V profile) and non-individualized programs (not considering the F-V profile).

The initial database search yielded a total of 868 studies. After that, 272 studies were excluded due to duplication and additional 585 based on the title and abstract screening. Eleven studies were assessed for full-text eligibility, while 6 of them were excluded due to the lack of reporting jump height or because they did not divide subjects into subgroups based on their F-V imbalance (i.e., force-deficit, velocity-deficit and well-balanced subgroup) or no non-individualized training program was set. Finally, five studies were included in this systematic review (Figure 1).

### Study Characteristics

A total of five studies, involving 217 participants (188 males, and 29 not reported) were included in this systematic review. Training programs lasted between 7 and 10 weeks, with an associated training session frequency of two to three per week. All studies involved jump height and F-V assessment prior to and following a training intervention. The age range of participants was from 16 to 28 years. The studies included participants majoring in rugby (N = 63), basketball (N = 30), ice-hockey (N = 16), handball (N = 14), soccer (N = 10), and professional futsal or semi-professional soccer and rugby (N = 84). Two studies were conducted during the mid-season (Jiménez-Reyes et al., 2017; Zabaloy et al., 2020), two during the pre-season (Barrera-Domínguez et al., 2023; Simpson et al., 2021), and one did not specify the time (Lindberg et al., 2021). The general characteristics of the studies are included in Table 1, while examples of training programs are provided in Table 2. The meta-analysis ultimately incorporated five studies, encompassing a total of 196 participants. The sub-group analysis was performed only in three studies (Jiménez-Reyes et al., 2017; Lindberg et al., 2021; Zabaloy et al., 2020) due to the fact that two studies included in the meta-analysis did not provide subgroup results (Barrera-Domínguez et al., 2023; Simpson et al., 2021).

**Table 1:** Summary of key characteristics of the sample, training volume, and jump types used in the studies included in the systematic review.

Reference	Participants	Age (years)	Training group (sample size)	Training frequency/ week (length)	Jump type
Jiménez-Reyes et al. (2017)	semi-professional male soccer & rugby players	23.1 ± 4.4	force-deficit (22) velocity-deficit (18) well-balanced (6) non-optimized (18)	2–3 (9 weeks)	SJ
Lindberg et al. (2021)	national male handball, ice-hockey & soccer players	20 ± 4	force-deficit (5) velocity-deficit (1) well-balanced (6) non-optimized (31)	2 (10 weeks)	SJ
Simpson et al. (2021)	professional rugby players	24 ± 3	optimized (15) non-optimized (14)	3 (8 weeks)	SJ
Zabaloy et al. (2020)	trained rugby players	n/a	force-deficit (6) velocity-deficit (11) well-balanced (9) non-optimized (8)	2 (7 weeks)	SJ
Barrera-Domínguez et al. (2023)	basketball players	22.9 ± 6.5	optimized (15) non-optimized (15)	2 (8 weeks)	CMJ

CMJ, countermovement jump; SJ, squat Jump; n/a, not specified; F-V, force-velocity

**Table 2:** Description of the individualized training programs based on the force-velocity (F-V) profile implemented in the different studies according to the detected F-V imbalance.

First Author (date)		Groups		Exercise		Sets/repetitions per set		Training Load	
Jiménez-Reyes et al. (2017)		High force-deficit		Back Squat Leg Press Deadlift Clean Pull SJ Single leg CMJ		3 sets each		80–90% 1RM 90–95% 1RM 90–95% 1RM 80% 1RM >70% BM 10% BM	
		Low force-deficit		Back Squat Leg Press Deadlift Clean Pull SJ Single leg CMJ		3 sets each		80–90% 1RM 90–95% 1RM 80% 1RM 80% 1RM 20–30% BM 10% BM	
		Well-balanced		Back Squat Deadlift SJ Single leg CMJ Depth Jumps Maximal Roller Push-Off		3 sets each		80–90% 1RM 80% 1RM 20–30% BM 10% BM BM <BM	
		Low velocity-deficit		Clean Pull Jump Single Leg CMJ SJ Depth Jumps Maximal Roller Push-Off CMJ with arms		3 sets each		65% 1RM 10% BM BM BM <BM BM	
		High velocity-deficit		Maximal Roller Push-off CMJ with arms Assisted SJ Depth Jumps CMJ SJ		3 sets each		<BM BM <BM BM 10%BM 20–30% BM	
Lindberg et al. (2021)		Force-deficit		Deadlift, Hip-thrust, Front squat, Squat, Stiff-leg deadlift, Bulgarian split squat, Calf-raises Trap Bar		7 (in total)/3–10 4/5		1–6 RIR (Reps in reserve) 50–70% 1RM	
		Well-balanced		Deadlift, Front squat, Bulgarian split squat, Hip-thrust, Deadlift Box jumps, Stair jumps, Single leg stair jumps, Squat jump w/rubber band, Stair jumps, Trapbar jumps		6 (in total)/3–10 7 (in total)/5–10		1–6 RIR Negative –50% 1RM	
		Velocity-deficit		Half Squat, Hip-thrust Squat jumps, Trapbar jumps, Step up, Squat jump w/rubber band, countermovement jumps, box jumps, Clean Pull, Stair jumps, Single leg stair jumps		3 (in total)/3–8 11 (in total)/5–10		1–2 RIR Negative–50% 1RM	
Simpson et al. (2021)		High force-deficit		Squat Box Squat Clean Pull Squat Jump Jump Shrug		n/a		>80% 1RM >80% 1RM 80% 1RM 75% 1RM 65% 1RM	
		Low force-deficit		Squat Box Squat Clean Pull Squat Jump Jump Shrug Squat Jump		n/a		>80% 1RM >80% 1RM 80% 1RM 70% 1RM 65% 1RM 20%–30% 1RM	
		Well-balanced		Squat Clean Pull Jump Shrug Squat Jump CMJ Depth Jump		n/a		>80% 1RM 80% 1RM 65% 1RM 20–30% 1RM 10% BM BM	
		Low velocity-deficit		Jump Shrug Squat Jump CMJ Squat Jump Depth Jump Accelerated Band Jump		n/a		65% 1RM 20%–30% BM 10% BM BM BM <BM	
		High velocity-deficit		Jump Shrug SJ CMJ Depth Jump Accelerated Band Jump		n/a		65% 1RM 10% BM BM BM BM <BM	
Zabaloy et al. (2020)		Velocity-deficit		Squat Squat Jump Unresisted Sprint		3 x 6 3 x 6 6 x 10 m		40% 1RM (40–60% BM)	
		Force-deficit		Squat Squat Jump Unresisted Sprint		3 x 6 3 x 6 6 x 10 m		75% 1RM (75–85% BM)	
		Well-balanced		Squat Squat Jump Unresisted Sprint		3 x 6 3 x 6 6 x 10 m		60% 1RM (60–75% BM)	
Barrera-Domínguez et al. (2023)		High force-deficit		Back Squat Deadlift trap Bar SL CMJ Deadlift Barbell Clean pull CMJ trap bar		0.68–0.51 m/s (10% VL) 0.71–0.58 m/s (10% VL) 4 reps 0.52–0.39 m/s (10% VL) >1.11 m/s (∼5% VL) 3 reps		80%–90% 1RM 70%–80% 1RM 10% BW 85%–95% 1RM 80% 1RM 80% BW	
		Low force-deficit		Back Squat CMJ trap bar SL CMJ Deadlift Barbell Clean pull SL SJ		0.68–0.51 m/s (10% VL) 3 reps 4 reps 0.52–0.39 m/s (10% VL) >1.11 m/s (∼5% VL) 4 reps		80%–90% 1RM 80% BW 10% BW 85%–95% 1RM 80% 1RM BW	
		Well-balanced		Back Squat Depth Jump SL CMJ Deadlift Barbell Clean pull Abalakov jump		0.68–0.51 m/s (10% VL) 5 reps 4 reps 0.71–0.58 m/s (10% VL) 5 reps 5 reps		80%–90% 1RM BW (30cm) 10% BW 70–80% 1RM 65% 1RM BW	
		Low velocity-deficit		Depth Jump SL SJ Band assisted CMJ Clean pull jump SJ Abalakov jump		5 reps 3 reps 5 reps 3 reps 3 reps 5 reps		BW (30cm) BW <BW 65% 1RM BW BW	
		High velocity-deficit		Band assisted CMJ Box Jump SL CMJ Abalakov jump CMJ Clean pull jump		5 reps 3 reps 3 reps 5 reps 3 reps 3 reps		<BW (30cm) BW BW BW 50% 1RM	

CMJ: Countermovement Jump, SJ: Squat Jump, 1RM: 1 Repetition maximum, RIR: Repetitions in Reserve, BW: bodyweight. m/s: metres/seconds, SL: Single Leg, VL: Velocity Loss

### Risk of Bias Assessment

The study of Lindberg et al. (2021) was found to have a high risk of bias in the selection of the reported results, while the other four studies showed some concerns in the randomization process (Barrera-Domínguez et al., 2023; Jiménez-Reyes et al., 2017; Simpson et al., 2021; Zabaloy et al., 2020). The remaining domains were assessed as having a low risk of bias for all other studies. A detailed individual assessment of bias, categorized as high, low, or some concerns, for each study is provided in Table 3.

**Table 3:** Risk of bias assessment.

Reference	Randomization process	Deviations from intended interventions	Missing outcome data	Measurement of the outcome	Selection of the reported result	Overall Bias
Jiménez-Reyes et al. (2017)	Some concerns	Low	Low	Low	Low	Some concerns
Lindberg et al. (2021)	Low	Low	Low	Low	High	High
Simpson et al. (2021)	Some concerns	Low	Low	Low	Low	Some concerns
Zabaloy et al. (2020)	Some concerns	Low	Low	Low	Low	Some concerns
Barrera-Domínguez et al. (2023)	Some concerns	Low	Low	Low	Low	Some concerns

### Meta-Analyses

#### F-V imbalance

**Figure 2:**
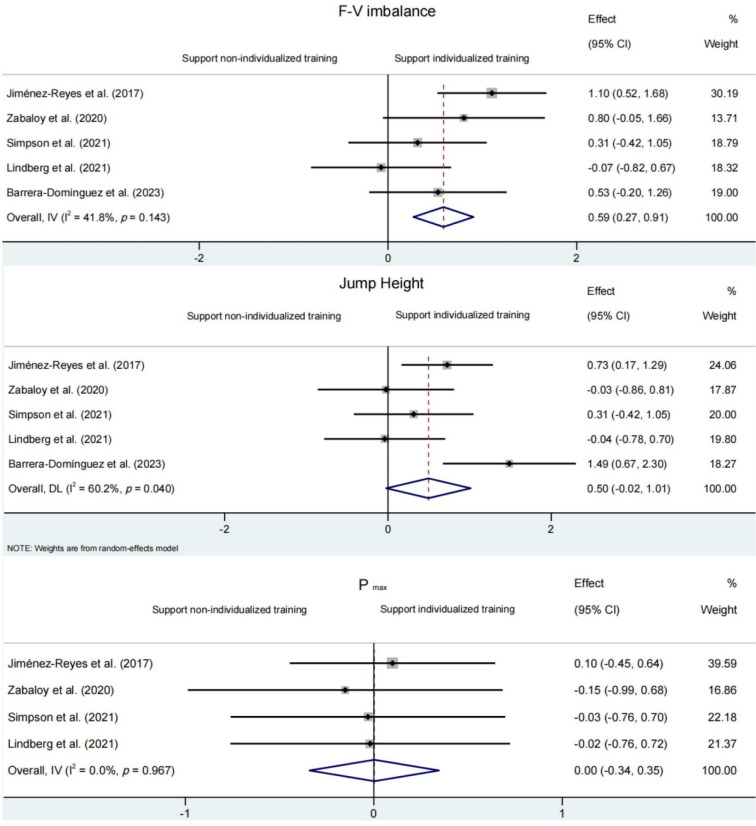
Comparison of changes in the force-velocity (F-V) imbalance (upper panel), jump height (middle panel), and maximal power (P_max_; lower panel) between individualized training programs (based on the force-velocity profile) and non-individualized training programs (not considering the F-V profile). The forest plot shows pooled standardized mean differences along with their 95% confidence intervals (CIs).

**Figure 3:**
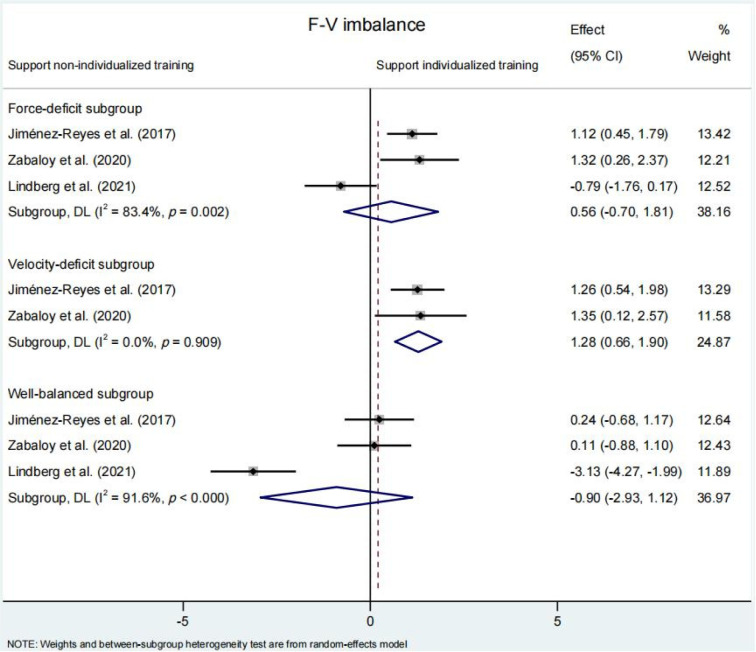
. Comparison of changes in the force-velocity (F-V) imbalance among three subgroups within the individualized training program based on the F-V profile (force-deficit, velocity-deficit, and well-balanced) compared to a non-individualized training program. Forest plot shows pooled standardised mean differences along with their 95% confidence intervals (CIs).

The reduction in the F-V imbalance was significantly greater following individualized compared to non-individualized training programs (SMD = 0.59 [95%CI: 0.27 to 0.91], *p* ≤ 0.001) (Figure 2). When compared against non-individualized training groups, participants belonging to the velocity-deficit subgroup experienced a significant decrement in F-V imbalances (SMD = 1.28 [95% CI: 0.66 to 1.90], *p*<0.001). However, this effect was not observed neither for the well-balanced subgroup (SMD = −0.90 [95% CI: −2.93 to 1.12], *p* = 0.382) nor for the force-deficit subgroup (SMD = −0.56 [95% CI: −0.70 to 1.81], *p* = 0.383) (Figure 3).

#### Jump Height

**Figure 4:**
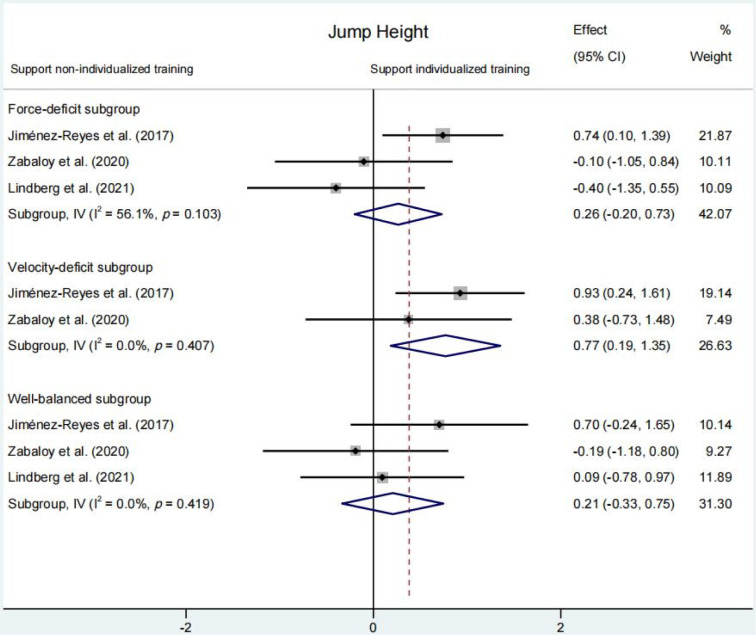
. Comparison of changes in jump height among three subgroups within the individualized training program based on the force-velocity profile (force-deficit, velocity-deficit, and well-balanced) compared to a non-individualized training program. Forest plot shows pooled standardised mean differences along with their 95% confidence intervals (CIs).

No significant differences were obtained for the change of jump height between individualized and non-individualized training programs (SMD = 0.50 [95% CI: −0.02 to 1.01], *p* = 0.059) (Figure 2). Similarly, no significant differences in changes of jump height were observed between the non-individualized group with a force-deficit (SMD = 0.26 [95% CI: −0.20 to 0.73], *p* = 0.684) and the well-balanced training subgroups (SMD = 0.21 [95% CI: −0.33 to 0.75], *p* = 0.451) (Figure 4). The only subgroup that experienced a significantly higher enhancement in jump height compared to non-individualized training groups was the velocity-deficit subgroup (SMD = 0.77 [95% CI: 0.19 to 1.35], *p* = 0.010) (Figure 4).

#### Maximal Power (Pmax)

**Figure 5:**
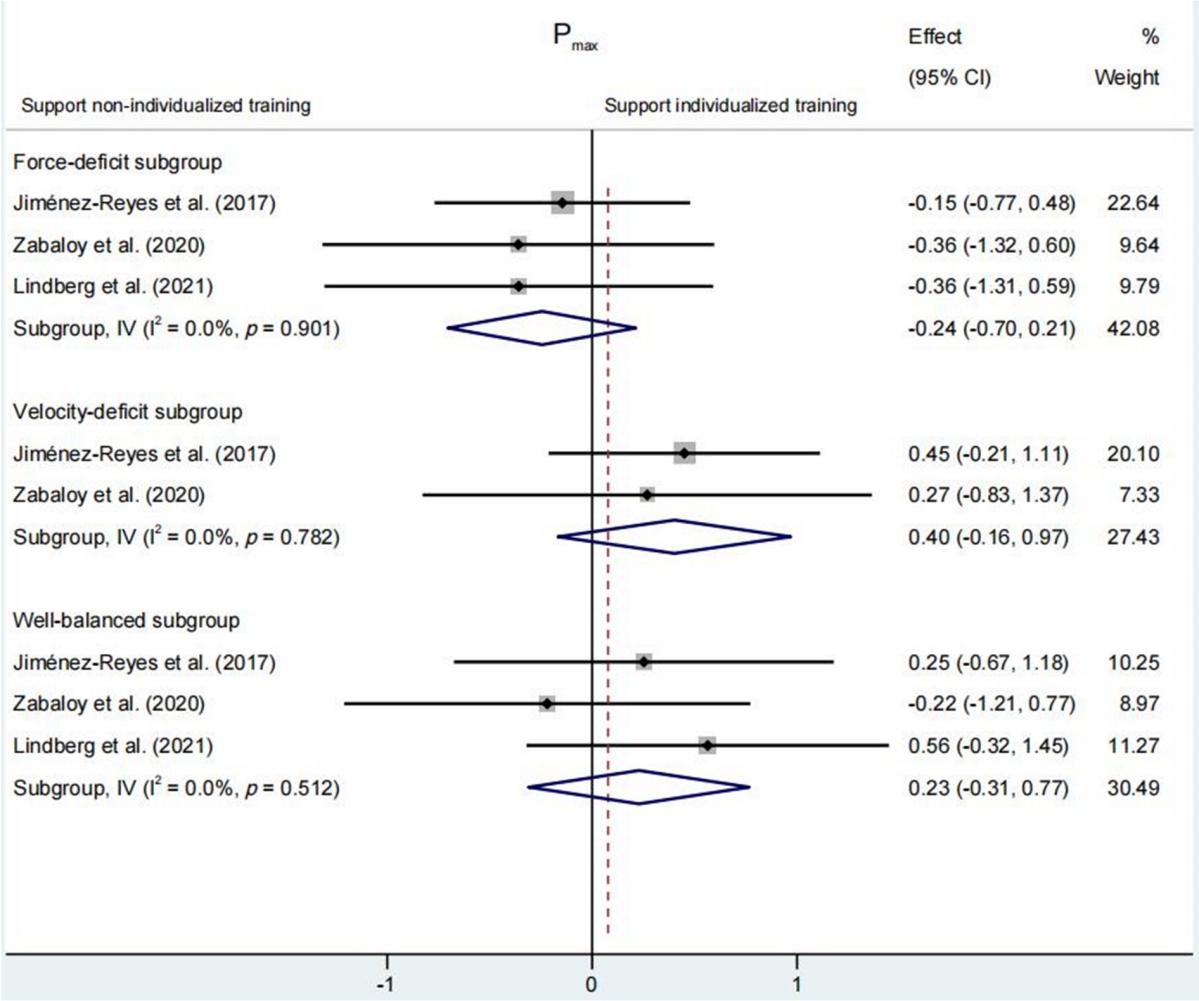
. Comparison of changes in maximal power (P_max_) among three subgroups within the individualized training program based on the force-velocity profile (force-deficit, velocity-deficit, and well-balanced) compared to a non-individualized training program. The forest plot shows pooled standardised mean differences along with their 95% confidence intervals (CIs).

No significant differences were found for P_max_ change between individualized and non-individualized training groups (SMD = 0.001 [95% CI: −0.34 to 0.35], *p* = 0.989) (Figure 2). Likewise, P_max_ change in the force-deficit subgroup (SMD = −0.24 [95% CI: −0.70 to 0.21], *p* = 0.296), the velocity-deficit subgroup (SMD = 0.40 [95% CI: −0.16 to 0.97], *p* = 0.165) and the well-balanced subgroup (SMD = 0.23 [95% CI: −0.31 to 0.77], *p* = 0.404) was comparable to that observed in the non-individualized group (Figure 5).

## Discussion

This systematic review with meta-analysis is the first to investigate whether individualized training programs based on the optimal F-V profile can effectively enhance jump height and P_max_ while reducing F-V imbalances. The findings suggest that while individualized training programs are effective in reducing F-V imbalances, they do not significantly improve jump height or P_max_ more than non-individualized training programs. Notably, only the velocity-deficit subgroup showed superior improvements in jump height compared to the non-individualized training program, whereas the force-deficit and well-balanced subgroups did not experience significant benefits compared to non-individualized training programs. Overall, these findings suggest that individualized programs based on the F-V profile may be particularly beneficial for athletes experiencing a velocity deficit. The present meta-analysis strongly supports the effectiveness of individualized training programs in reducing F-V imbalances.

Unlike non-individualized training programs, the individualized training methodology targets specific parts of the F-V continuum by selecting heavy and light training loads that more directly impact force and velocity capacities, respectively. This approach allows for desired alterations in the F-V profile, potentially leading to enhancements in P_max_ and jump height (Jiménez-Reyes et al., 2019; Samozino et al., 2014). Specifically, this is done by applying heavy load resistance exercises (>80% 1RM) such as squats, deadlifts, and clean pulls for athletes presenting force deficit, while light (< 30% 1RM) or negative loads power exercises and plyometric training exercises are used by athletes experiencing velocity deficit (Table 2). However, if the correct intensities (i.e., loads) are not used in training protocols, the effectiveness of the individualized training programs diminishes or vanishes. For example, in the study by Lindberg et al. (2021), the loads ranged from 50 to 70% of 1RM for force-deficit athletes and less than 50% of 1RM for velocity-deficit athletes, while the weekly training volume was lower than in other studies (i.e., 15 sets vs. 18 sets). Therefore, changes in F-V imbalances and performance variables were expected to be influenced not only by the use of individualized or non-individualized training programs, but also by the selection of appropriate training intensities and volumes.

Athletes following individualized training programs did not significantly outperform athletes belonging to non-individualized training groups in terms of jump height improvement, although a moderate effect size in favor of the individualized training group was observed (SMD = 0.50). A potential explanation for the results not favoring individualized training programs when it comes to jump height enhancement could be an inappropriate selection of training loads and volumes in one out of five studies, which surprisingly also failed to reduce F-V imbalances (Lindberg et al., 2021). The failure to reduce the F-V imbalance was surprising given the velocity- specific training principle, which states that training at specific velocities should accentuate performance improvements at those velocities. Another explanation might be attributed to the similarity of some exercises between groups. For example, employing a light loaded squat jump (SJ) for the velocity-deficit subgroup might not be ideal, as this exercise is positioned closer to *F_0_* rather than *v_0_* (around 60% of *F_0_* and 40% of *v_0_* for athletes with a force deficit at 30% of 1RM in squat jumps). Considering the remaining four studies, the superiority of individualized training programs over non-individualized ones in enhancing jump height was supported (SMD = 0.64, [95% CI: 0.28 to 0.99], *p* < 0.001) (Barrera-Domínguez et al., 2023; Jiménez-Reyes et al., 2017; Simpson et al., 2021; Zabaloy et al., 2020). Additionally, two studies investigated the effects of individualized training based on the SJ F-V relationship while also assessing changes in CMJ height, both yielding results similar to those observed for the SJ (Lindberg et al., 2021; Zabaloy et al., 2020). These results collectively indicate the importance of prescribing a training program that effectively reduces the F-V imbalance to further enhance jump height.

No significant differences in P_max_ were observed between athletes following an individualized training program and those following non-individualized ones. One interesting training design was found in the study by Barrera-Domínguez et al. (2023). The potential advantage of the study by Barrera-Domínguez et al. (2023) lies in limiting the number of repetitions in a set by applying a 10% velocity loss threshold. Compared to other protocols, the 10% velocity loss threshold has been shown to effectively reduce fatigue and improve the rate of force development and power output in the lower limbs (Jukic et al., 2022). Additionally, Barrera-Domínguez et al. (2023) included supplementary plyometric training for all subgroups, not just for velocity-deficit subgroups, which may further inspire the design of individualized training. Future research should investigate whether implementing some of velocity-based strategies (e.g., velocity loss thresholds for fatigue control) and additional plyometric training can significantly impact P_max_ improvement. The greater positive impact of the individualized training program on jump height compared to P_max_ aligns with the initial proposal by Samozino and colleagues (2012) who argued that jump height could be improved even without changes in P_max_ when the disparities between the actual and optimal F-V profiles were reduced (Samozino et al., 2012).

The velocity-deficit subgroup was the only subgroup where athletes exhibited a decrease in the F-V imbalance followed by jump height improvement while maintaining similar P_max_ compared to the non-individualized group. Nevertheless, these findings should be interpreted with caution due to the limited number of studies included in this analysis and potential problems in training methodology applied in some studies. A possible explanation for not finding significant decrements in F-V imbalances in the force-deficit subgroup might be the low reliability of the F-V profiles generally reported in the literature (Kotani et al., 2022; Lindberg et al., 2021; Valenzuela et al., 2021). Although Samozino et al. (2022) suggested that F-V profiles were reliable when the variability in the push-off distance and jump height was lower than 5%, some studies found this difficult to achieve in practice (Li et al., 2023; Lindberg et al., 2021). Therefore, selecting the appropriate training stimulus to induce specific changes in the F-V profile and ensuring that the F-V profile is measured with high reliability are essential when implementing individualized training programs (Zabaloy et al., 2025; Zhang et al., 2025).There are several limitations to consider when interpreting the results of this review. First, the majority of participants in this systematic review and meta-analysis were male, young, trained athletes, which can potentially restrict the applicability of our findings to other demographics, such as females, older individuals and those who are untrained. However, since obtaining a reliable vertical F-V profile requires individuals to jump against a wide range of external loads, this training methodology may only be valuable for strong athletes. Second, our results on the subgroup effectiveness of individualized training programs are limited, as only three out of five studies categorised subjects into force-deficit, velocity-deficit, and well- balanced subgroups. Third, the present study's results only reflect the effectiveness of individualized training programs on the SJ and the CMJ, while it would be interesting to investigate their impact on jump height in more complex jumps. Finally, not all studies included in the meta-analysis stratified participants into subgroups based on low (i.e., F-V profile deviates up to 40% from the optimal) and high (i.e., F-V profile deviates more than 40% from the optimal) force or velocity deficits. This lack of stratification may have influenced the outcomes, as individuals with greater deficits might derive more significant benefits from individualized training programs compared to those with lower deficits (Samozino et al., 2014b).

## Conclusions

Individualized training programs were more effective at reducing F-V imbalances than non-individualized programs. This result can be explained by the velocity-specific training principle, as individualized training programs target specific segments of the F-V profile. However, individualized training programs did not lead to superior improvements in jump height or P_max_. This outcome might be influenced by studies that failed to induce specific shifts in the F-V profile following force-deficit and velocity-deficit training. Despite not reaching statistical significance, individualized training programs appear to be more favorable for jump height compared to P_max_, supporting the notion that jump height can be improved without changes in P_max_ by reducing disparities between actual and optimal F-V profiles. Future studies should adopt a consistent approach when grouping participants for individualized training, ensuring that groups consist of a similar number of participants with comparable magnitude of force and velocity deficits to accurately assess the potential of individualized training programs on vertical jump performance.
